# A Strategy for Hydroxide Exclusion in Nanocrystalline Solid-State Metathesis Products 

**DOI:** 10.3390/nano3030317

**Published:** 2013-06-24

**Authors:** Jiaqi Cheng, Kristin M. Poduska

**Affiliations:** 1 Department of Chemistry, Memorial University of Newfoundland, St. John’s, NL A1B3X7, Canada; E-Mail: jiaqic@mun.ca; 2 Department of Physics and Physical Oceanography, Memorial University of Newfoundland, St. John’s, NL A1B3X7, Canada

**Keywords:** solid-state synthesis, metathesis, nanoparticles, X-ray diffraction, vibrational spectroscopy

## Abstract

We demonstrate a simple strategy to either prevent or enhance hydroxide incorporation in nanocrystalline solid-state metathesis reaction products prepared in ambient environments. As an example, we show that ZnCO_3_ (smithsonite) or Zn_5_(CO_3_)_2_(OH)_6_ (hydrozincite) forms extremely rapidly, in less than two minutes, to form crystalline domains of 11 ± 2 nm and 6 ± 2 nm, respectively. The phase selectivity between these nanocrystalline products is dominated by the alkalinity of the hydrated precursor salts, which may in turn affect the availability of carbon dioxide during the reaction. Thus, unlike traditional aqueous precipitation reactions, our solid-state method offers a way to produce hydroxide-free, nanocrystalline products without active pH control.

## 1. Introduction

Nanoparticle production via solid-state synthesis often involves metathesis of well-mixed solid precursors that react exothermically and quickly through a well-known class of self-sustaining reactions [1]. In comparison with traditional sol-gel or solvo-thermal routes, the absence of solvent in solid-state metathesis (SSM) has enabled rapid formation of a wide range of materials including metal oxides [2,3,4,5], sulfides [6], perovskites [7], and zeolites [8]. A typical synthesis is carried out at ambient temperature, pressure, and atmosphere conditions, where two powdered precursors are ground together. Once the reaction is triggered, a self-sustained exothermic reaction proceeds: no external heating is required. This metathesis is driven by thermodynamics and the formation of stable crystal products. Although the mechanism for ambient SSM is still not completely understood, some have noted that there is a class of SSM reactions that appears to benefit from the precursors’ waters of hydration and/or from ambient water that is adsorbed at precursor grain interfaces. It is possible that this small amount of water, which is released during the exothermic reaction to make a slurry with the starting powders, promotes diffusion and lowers the reactions’ activation energies [6]. 

Although ambient SSM has opened up a new window for expedient and solvent-free synthetic routes for many technologically and industrially relevant nanomaterials, unwanted incorporation of hydroxide or CO_2_ species compromise the purity of the final product. These secondary products cannot always be removed by rinsing with water or organic solvents, and they are sometimes best removed by high temperature calcination [7]. Some reports have shown that this problem can be mitigated by dosing the precursor mixture with surfactants or other additives to control both crystal habit and composition [4,9]. However, these additional components can present different issues for product purification, and the role of additives with regard to composition control during the metathesis process remains unclear. 

Herein, we demonstrate that we can control hydroxide incorporation in nanocrystalline products using ambient SSM with a careful selection of precursors to affect pH in the small amount of water that is present. We demonstrate the efficacy of this approach with zinc carbonate nanocrystalline products: the hydroxide-free ZnCO_3_ (ZC, smithsonite), as well as hydrozincite, Zn_5_(CO_3_)_2_(OH)_6_ (HZ). In nanocrystalline form, zinc carbonates have found industrial use as surface-active absorbers in respirators for health and safety applications [10]. Neither additives nor post-synthesis annealing are required to regulate the composition of the metathesis product, nor is there any active pH regulation required during the synthesis. 

## 2. Experimental Section

**Figure 1:**
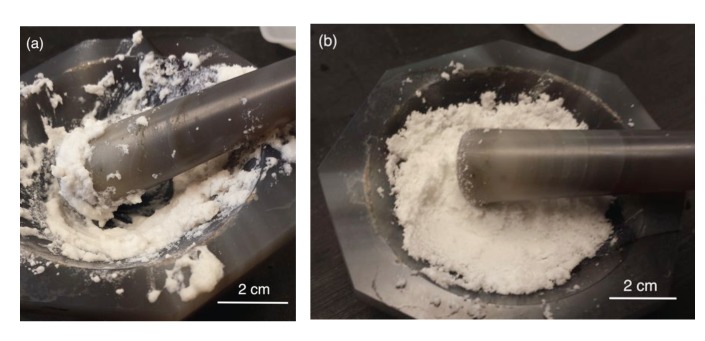
Representative photographs of the paste-like reaction products from (**a**) Zn(NO_3_)_2_·6H_2_O and NaHCO_3_ precursors, which yields ZnCO_3_; and (**b**) ZnCl_2_ + Na_2_CO_3_, which yields Zn_5_(CO_3_)_2_(OH)_6_.

Our experiments began with analytical grade reagents: 0.5 mol of Zn(NO_3_)_2_·6H_2_O was mixed with 1.5 mol of NaHCO_3_ and then mixed throughly by hand in an agate mortar. The importance of this mixing is not at all related to mechanical pressure; instead, it is the intermingling of the powdered precursors that triggers the reaction. The total mass of the precursors was typically ∼1 g, but scaling up the reaction by a factor of ten did not adversely affect the results. After about 1 minute of mixing, the exothermic reaction within the mixture yielded a wet white paste, with only a small temperature increase (∼5 °C). Photographs of the paste-like products are shown in Figure 1. This product was transferred to anhydrous ethanol and washed several times with ultrapure water (18.2 MΩ·cm) to remove soluble ions. The remaining product was oven dried at 80 °C for at least 24 h, prior to further characterization. Similar experiments were repeated with different combinations of precursors, as shown in Table 1. Results are highly reproducible, and were tested at least three times for each combination, including slight variations in the reactant ratios (0.67:1, 1:1, 1.5:1). 

**Table 1:** Products from ambient SSM using different precursor combinations. Here, HZ means Zn_5_(CO_3_)_2_(OH)_6_ and ZC means ZnCO_3_. Gibbs free energy data are from [11].

Precursors	Product	Calc. ZC ΔG_0_ (kJ/mol)	Calc. HZ ΔG_0_ (kJ/mol)
		at 25 °C	at 25 °C
ZnCl_2_ + NaHCO_3_	none	−64	−278
ZnCl_2_ + Na_2_CO_3_	HZ	−86	−391
Zn(NO_3_)_2_·6H_2_O + Na_2_CO_3_	HZ	−71	−483
Zn(NO_3_)_2_·6H_2_O + NaHCO_3_	ZC	−85	−203

All samples were characterized by powder X-ray diffraction (PXRD; Ultima IV X-ray diffractometer (Rigaku, Texas, U.S.A.) with Cu Kα 3°/min, step size 0.02°; lattice constant refinements from Jade software, Materials Data Inc. (Livermore, CA, U.S.A.) and compared with JCPDS data [12]), Fourier transform infrared spectroscopy (FTIR; Alpha spectrometer (Bruker, Billerica, MA, U.S.A.) at 4 cm^−1^ resolution on specimens dispersed in a 7 mm diameter KBr pellet), and Raman spectroscopy (Renishaw inVia Raman microscope, 830 nm excitation). Thermal decomposition experiments were conducted with a Q500 thermogravimetric analyzer (TA Instruments, New Castle, DE, U.S.A.) using a Pt pan, 600 °C, 20.00 °C/min, under 40.0 mL/min N_2_ gas flow). Crystalline domain sizes were extracted by Scherrer analyses on PXRD peak widths using at least 11 diffraction peaks. This approach gives a truly representative average crystalline domain size, since the data were obtained using ∼1 g of powder. Brunauer-Emment-Teller (BET) analyses provided surface area values. 

## 3. Results and Discussion

**Figure 2:**
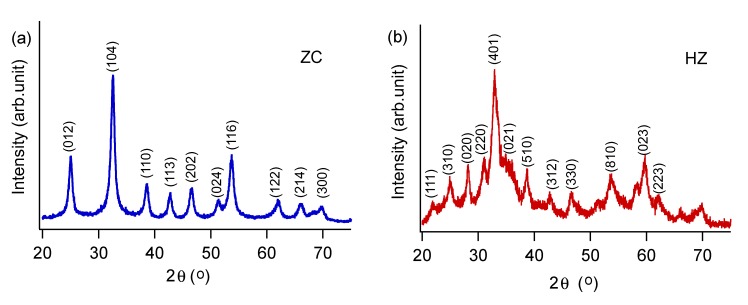
Representative indexed X-ray diffraction (XRD) data. In (**a**), all major peaks corresponding to ZnCO_3_ (JCPDS 8-0449) are present when starting with Zn(NO_3_)_2_ and NaHCO_3_ precursors. In (**b**), the product matches Zn_5_(OH)_6_(CO_3_)_2_ (JCPDS 19-1458).

**Figure 3:**
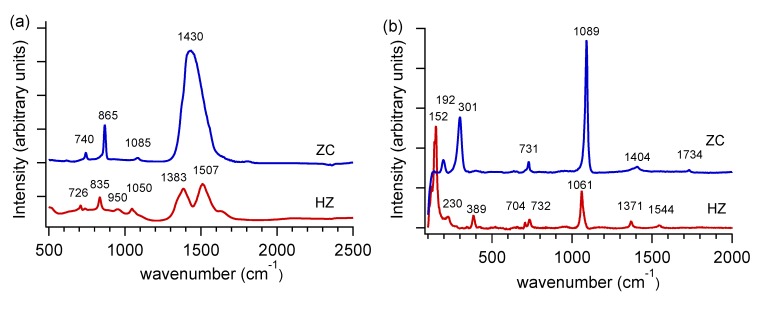
Representative (**a**) Fourier transform infrared spectroscopy (FTIR) spectra and (**b**) Raman spectra for ZnCO_3_ (ZC, blue) and Zn_5_(CO_3_)_2_(OH)_6_ (HZ, red). Spectra are offset along the vertical axis for clarity.

The particles in the product are truly nanocrystalline, with crystalline domain sizes of 11 ± 2 nm for ZC and 6 ± 2 nm for HZ, and surface areas of 25.4 ± 0.3 m^2^/g. From PXRD data, we find that ZC has sharper diffraction peaks (Figure 2a), while HZ occurs with poorer crystallinity (Figure 2b). For this reason, we also used FTIR and Raman spectroscopies to corroborate the phase compositions of the products. As shown in the representative FTIR spectrum in Figure 3a, ZC displays an intense single peak at 1430 cm^−1^ (*ν*_3_ antisymmetric carbonate stretch). Hydroxide incorporation in HZ lowers the symmetry of this carbonate vibration [13] to yield multiple peaks between 1380 and 1510 cm^−1^.A low intensity peak near 1085 cm^−1^ (*ν*_1_ carbonate stretch) is IR inactive, according to ideal symmetry considerations [14], but is very intense in Raman spectra (Figure 3b). For HZ, this *ν*_1_ mode is not observed in either FTIR or Raman spectra, however, the presence of a peak at 950 cm^−1^ has been attributed to a Zn-OH distortion in previous reports [15]. The carbonate *ν*_2_ and *ν*_4_ bending modes cause sharp peaks at 865 cm^−1^ and 740 cm^−1^, respectively, in ZC; these peaks shift to lower wavenumbers and broaden in HZ. 

**Figure 4:**
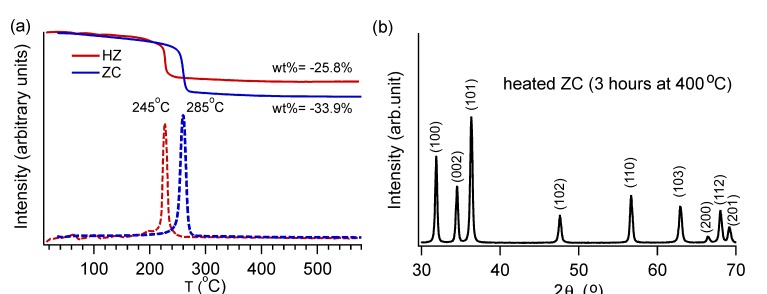
(**a**) ZnCO_3_ (ZC, blue) and Zn_5_(CO_3_)_2_(OH)_6_ (HZ, red) show one-step decomposition toward the formation of ZnO. The mass loss curves are shown as solid lines, and their derivatives are shown as dashed lines; (b) The decomposition products match ZnO (JCPDS 36-1451). The representative data shown here are for the product from ZC decomposition.

We performed additional experiments to ensure the SSM reactions yield phase-pure products. Thermal decomposition data indicate that both ZC and HZ undergo a one-step decomposition process, releasing their carbonate and hydroxide ions simultaneously (Figure 4). The theoretical mass loss [16] for ZC is 35.1% when ZnCO_3_ → ZnO + CO_2_; our yield was 33.9% ± 0.5%. For HZ, we measured a mass loss of 25.8% ± 0.5%, which agrees well with the expected value [17] of 25.9% based on the reaction Zn_5_(CO_3_)_2_(OH)_6_ → 5ZnO + 2CO_2_ + 3H_2_O. PXRD measurements confirmed that the decomposition product was ZnO, consistent with decomposition studies reported by others [18,19,20]. 

By comparing combinations of different precursors (Table 1), we find that only Zn(NO_3_)_2_·6H_2_O + NaHCO_3_ yields the hydroxide-free ZnCO_3_. Based on results of previous aqueous precipitation experiments [10,13,17,21,22,23,24], it appears that NaHCO_3_ provides sufficient acidity and CO_2_ to produce ZC, while Na_2_CO_3_ does not. We note that other carbonate precursor salts can also be used with similar effect. For example, K_2_CO_3_ gives results that are identical to those with a Na_2_CO_3_ precursor. It is also evident that the choice of the Zn precursor is quite important. For example, a self-sustained reaction did not occur between ZnCl_2_ and NaHCO_3_. We note that the wettest slurry occurred when using Zn(NO_3_)_2_·6H_2_O and NaHCO_3_ (as shown in Figure 1a). This would suggest that having sufficient water, contributed by either hydration waters or by absorption from the ambient environment, is also important to enable a self-sustained SSM reaction in this system. 

To assess the thermodynamics of the different precursor reactions shown in Table 1, we calculated the Gibbs energy of reaction based on the following four equations, in which X is either NO_3_^−^ or Cl^−^ and n is an integer:

(1) 5 · ZnX_2_ · *n*H_2_O + 5 · Na_2_CO_3_ → Zn_5_(CO_3_)_2_(OH)_6_ + 3 · CO_2_ + 10 · NaX + (5*n* − 3) · H_2_O

(2) ZnX_2_ · *n*H_2_O + Na_2_CO_3_ → ZnCO_3_ + 2NaX + *n* · H_2_O

(3) ZnX_2_ · *n*H_2_O + 2 · NaHCO_3_ → ZnCO3 + CO_2_ + (*n* + 1) · H_2_O + 2 · NaX

(4) 5 · ZnX_2_ · *n*H_2_O + 10 · NaHCO_3_ → Zn_5_(CO_3_)_2_(OH)_6_ + 8CO_2_ + (5*n* − 3) · H_2_O + 10 · NaX

In all four cases, the negative Gibbs energy of reaction values indicate that the reactions can proceed spontaneously. 

Both of our zinc carbonate products (ZC and HZ) have a low solubility in water and, as a consequence, aqueous syntheses by direct precipitation have been reported for each phase using a variety of different zinc salt precursors, including ZnCl_2_, Zn(NO_3_)_2_, ZnSO_4_, and Zn(CHCOO)_2_. HZ is the most common product under standard temperature and atmosphere conditions [21,22,23], with ZC formation reported only with acidic pH control [17] high CO_2_ pressure [13,24], or low temperatures [10]. In contrast, there is only one report of phase-pure ZC through ambient SSM, in which the authors conclude that the precursors (NH_4_HCO_3_ and ZnSO_4_) had to be ground in the presence of an additional surfactant (polyethyleneglycol-octyl-phenylate) in order to yield ZC; a mechanism was not proposed [9]. 

We propose that three factors must be met for selective ZC or HZ production: negative Gibbs free energy of reaction, sufficient structural and/or surface water, and crude pH control. As shown in Table 1, the calculated standard Gibbs free energy of reaction for each salts combination in SSM are all negative and thus thermodynamically possible. The Na_2_CO_3_ precursor, we believe, contributes to hydroxide incorporation in the product due to CO_3_^2−^ hydrolysis. In contrast, the dissociation of NaHCO_3_ during the exothermic reaction provides a ready source of gaseous CO_2_ that can be dissolved in the reaction slurry. HCO^3−^ also provides a sufficiently acidic environment in the slurry by preventing hydrolysis of CO_3_^2−^ with ambient water. 

## 4. Conclusions

It is surprising—and very widely applicable—that a solid-state synthesis method can be adjusted to provide a robust way to exclude hydroxide under ambient temperature and atmosphere conditions. Crude pH control during the reaction is provided by acid-producing, hydrated precursor salts, and this is sufficient to produce phase-pure ZnCO_3_. This is in stark contrast to reports of aqueous-based precipitation reactions for ZnCO3, which have shown that accurate pH control is essential—acidic pH values (≤6) lead to ZC dissolution, while excessively alkaline conditions promote Zn(OH)_2_ formation at the expense of ZC [10,13,17,24]. Furthermore, our solid-state products are nanocrystalline, in both the ZC and HZ forms, due to the extremely rapid formation process. Our strategy of using acid-producing salts to form hydroxide-free carbonates could likely be extended to other classes of compounds for which pH is a tuning parameter for phase selectivity. 
